# A new species of *Chrysosplenium* (Saxifragaceae) from Northeastern China

**DOI:** 10.3897/phytokeys.135.39036

**Published:** 2019-11-25

**Authors:** Yong-In Kim, Jae-Seo Shin, Sangwoo Lee, Jia-Hui Chen, Sangho Choi, Jin Hee Park, Young-Dong Kim

**Affiliations:** 1 Department of Life Sciences, Multidisciplinary Genome Institute, Hallym University, Chuncheon 24252, South Korea Hallym University Chuncheon South Korea; 2 CAS Key Laboratory for Plant Diversity and Biogeography of East Asia, Kunming Institute of Botany, Chinese Academy of Sciences, Kunming, Yunnan 650201, China Korea Research Institute of Bioscience and Biotechnology Daejeon South Korea; 3 International Biological Material Research Center, Korea Research Institute of Bioscience and Biotechnology, Daejeon 34141, South Korea CAS Key Laboratory for Plant Diversity and Biogeography of East Asia Kunming China; 4 Freshwater Bioresources Research Division, Nakdonggang National Institute of Biological Resources, Sangju 37242, South Korea Nakdonggang National Institute of Biological Resources, Sangju South Korea

**Keywords:** Saxifragales, seed morphology, sterile branch, taxonomy

## Abstract

This study describes and illustrates *Chrysosplenium macrospermum* Y.I.Kim & Y.D.Kim, a new plant species from Changbaishan Mt. (Baekdusan Mt.) in northeastern China. The species is most similar to *Chrysosplenium valdepilosum* in the series *Pilosa* but is readily distinguishable by short arching sterile branches, multiple (up to 3) flowering stems, and smooth surfaced seeds (without tubercles), which are ca. 30–50% larger than those of other members in the series.

## Introduction

*Chrysosplenium* L. (Saxifragaceae) is a genus of small succulent and fragile herbs characterized by tetramerous flowers with petaloid sepals (Bensel and Palser 1975, Soltis 2007, Soltis et al. 2001). It is composed of approximately 70 species, mainly distributed in temperate regions of the Northern Hemisphere, except for two disjunctive species in Chile (Hara 1957, Spongberg 1972, Pan 1986, Ye and Zhang 1994, Wakabayashi and Takahashi 1999, Han and Kang 2012, Bhaumik 2014, Kim and Kim 2015, Liu et al. 2016, Kim et al. 2018, Wakabayashi et al. 2018). They usually exhibit dramatic morphological changes in the shapes of sterile branches during flowering and fruiting periods, and variations in size depending on the environmental conditions (e.g., humidity, light). For these reasons, correct identification and species delimitation have been the most challenging taxonomic tasks in relation to this genus. More detailed and comprehensive morphological studies encompassing various developmental periods have led to the discovery of five new *Chrysosplenium* species over the past five years (Bhaumik 2014, Kim and Kim 2015, Liu et al. 2016, Kim et al. 2018, Wakabayashi et al. 2018).

Recently, molecular phylogenetic approaches have provided valuable assistance in the effort to detect cryptic lineages in many plant groups, including *Chrysosplenium*. During an ongoing phylogenetic study of *Chrysosplenium* series *Pilosa* Maxim., we came across a new taxon that was collected near Tianchi Crater Lake in Changbaishan, Jilin, in China. Additional fieldwork was conducted in July 2017 to collect flowering individuals and seeds for more detailed morphological examinations. After a comprehensive examination of herbarium specimens (at HHU, TI, KB, KH, KWNU, KUS, IUI, KYO, and PE and at the Global Plants website of JSTOR) and literature related to *Chrysosplenium* (Franchet and Savatier 1878, Nakai 1914, Kitagawa 1934, Ohwi 1934, Hara 1957, Pan 1986, Pan and Ohba 2001, Han and Kang 2012, Kim and Kim 2015, Kim et al. 2018), we recognized that the taxon is a new species and belongs to the series *Pilosa*. Here, the new species is described and illustrated.

## Materials and methods

Photographs of the plant habit and macro-morphological characters were taken in the field. Morphological observations and measurements of the new species were conducted based on living and dried specimens and preserved materials. All morphological characters were observed and photographed with a Zeiss Stemi SV 11 Apo stereoscopic microscope and a Zeiss AxioCam MRc 5 microscope camera. Seed coat characters were examined by a Hitachi S-3400N scanning electronic microscope.

## Taxonomic treatment

### Chrysosplenium macrospermum

Taxon classificationPlantaeSaxifragalesSaxifragaceae

Y.I.Kim & Y.D.Kim sp. nov.

83C2D073-0C17-5187-8F13-98AE0860CC92

urn:lsid:ipni.org:names:77203161-1

**Figure 1.**
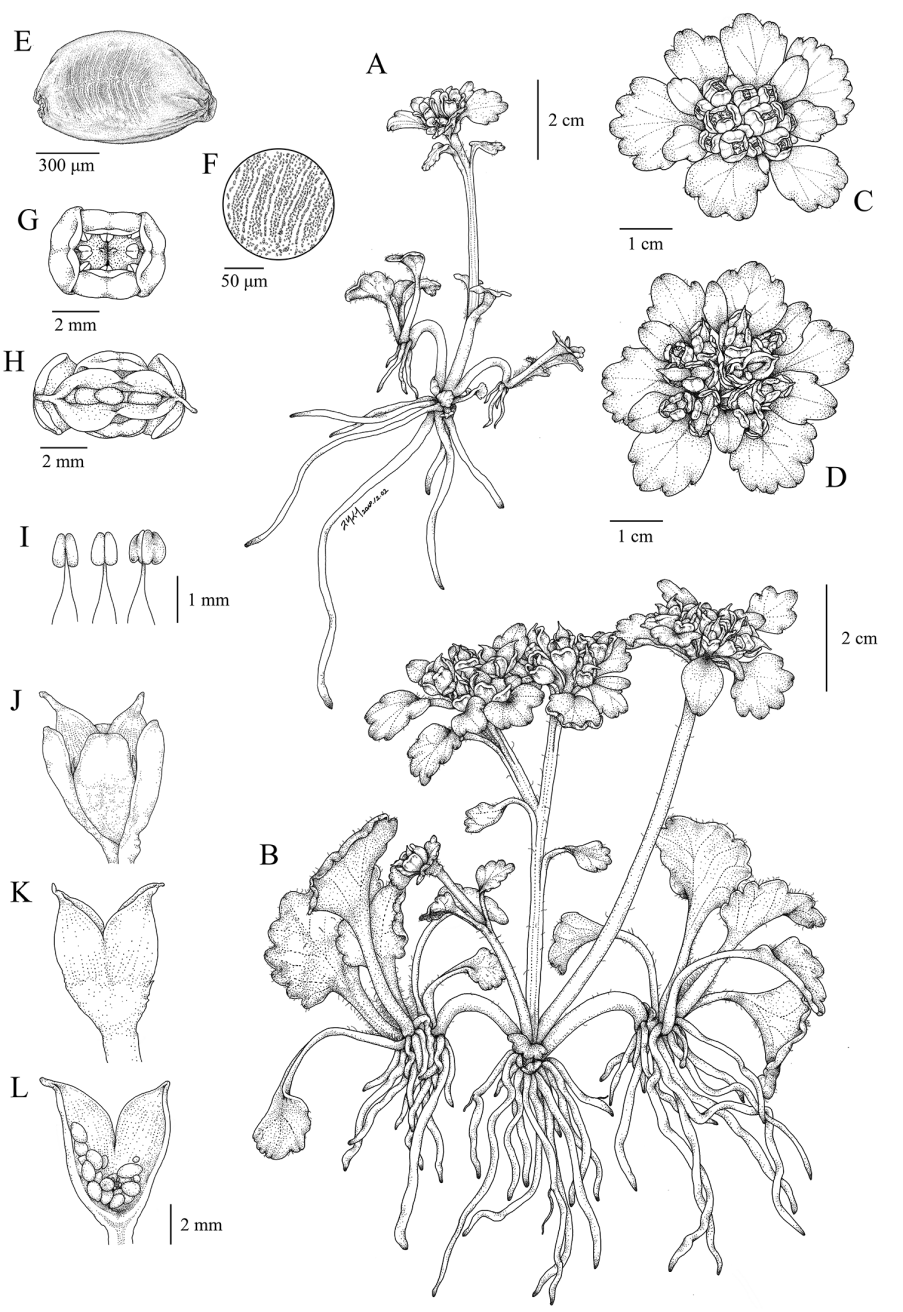
*Chrysosplenium macrospermum* Y.I.Kim & Y.D.Kim, sp. nov. **A** flowering individual **B** fruiting individual **C** inflorescence and bracteal leaves **D** infructescence and bracteal leaves **E** seed **F** seed coat, enlarged **G** flower (top view) **H** capsule, after dehiscence (top view) **I** stamen at various stages **J** capsule with persistent sepals (side view) **K** capsule, sepals removed **L** capsule, longitudinal section.

**Figure 2.**
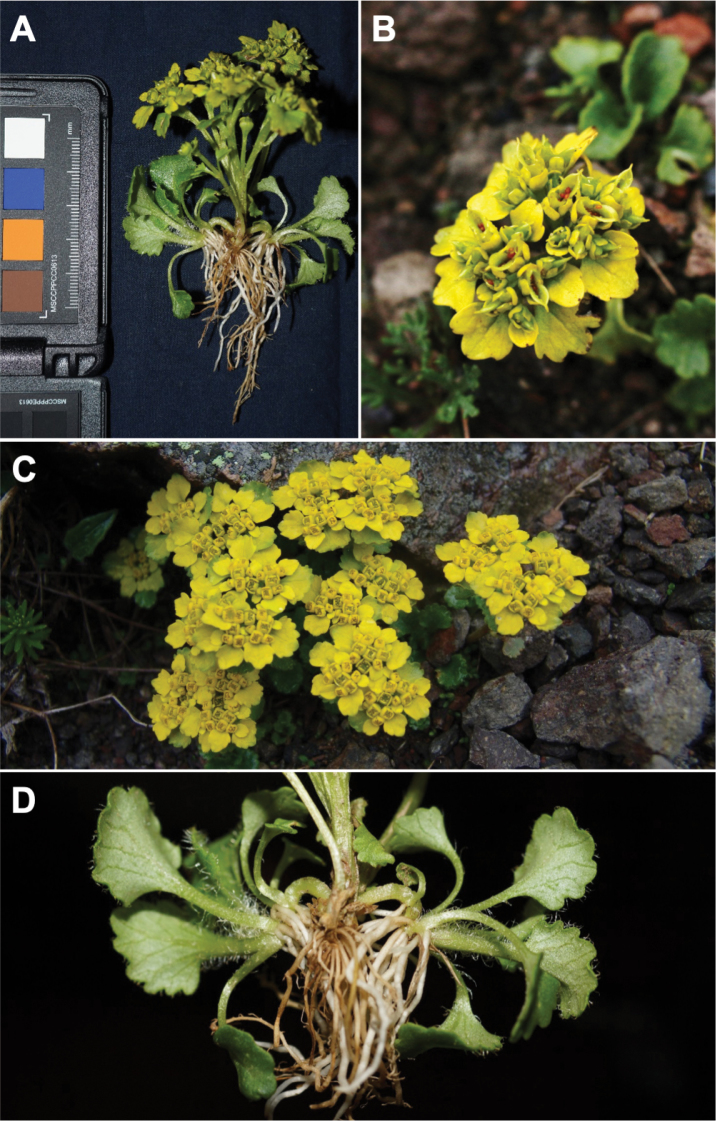
*Chrysosplenium macrospermum* Y.I.Kim & Y.D.Kim, sp. nov. **A** fruiting individual **B** infructescence, bracteal leaves and seeds in capsules **C** plant habit during flowering **D** fruiting individual showing short arch-shaped sterile branches and thick fibrous roots.

**Figure 3.**
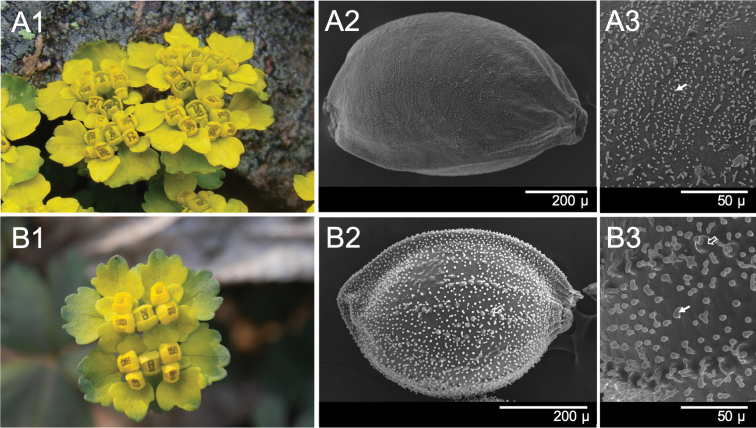
*Chrysosplenium* spp. inflorescence and seeds. **A***C macrospermum* Y.I.Kim & Y.D.Kim, sp. nov., inflorescence with bracteal leaves (**A1**), seed surface, scanning electron micrograph, 140× (**A2**) and 600× (**A3**) **B***C. valdepilosum*, inflorescence with bracteal leaves (**B1**), seed, scanning electron micrograph, 350× (**B2**) and 600× (**B3**). White solid arrows indicate deciduous papilla (**A3, B3**) and blanked arrows indicate tubercle (**B2, B3**).

Figs 1 , 2 , 3A1, A2

#### Diagnosis.

*Chrysosplenium macrospermum* is most similar to *Chrysosplenium valdepilosum* (Ohwi) S.H. Kang & J.W. Han, 2011 (see Han et al. 2011), but the former is readily distinguishable by short arching sterile branches, multiple (up to 3) flowering stems, and smooth surfaced seeds (without tubercles), which are ca. 30–50% larger than those of other members in the series *Pilosa* (Figure 3).

#### Type.

China. Jilin: near Tianchi (Cheon-Ji in Korean) Crater Lake to Changbaishan Mt. (Beakdusan Mt. in Korean), Antu County, Changchun, 42°01'44.80"N, 128°03'59.22"E, elev. 2,610 m, 26 Jul. 2017, *KYI-2017001* (holotype HHU; isotypes HHU, KB, KRIB).

#### Description.

Perennial herbs. Small (up to 7 cm), hermaphroditic. Roots thick fibrous. Flowering stem(s) 1–3, erect, 2–7 cm long, sometimes branched, tetragonal in the cross-section, sparsely pilose along the edges, light green to green, with 2(3) sterile branches arising from the base; sterile branches 1–1.5 cm long, stout, arch-shaped, sparsely pilose. Leaves simple, estipulate, petiolate. Basal leaves (1) or 2, opposite, petiole 3–15 mm long, blade up to ca. 1 × 1 cm, flabelliform. Cauline leaves of flowering stem(s) 1–4, opposite or rarely alternate, attached at 1/2 or below of the stem; petiole 1–10 mm long, entirely ciliate; blade 2–10 × 3–11 mm, flabelliform, apex subtruncate to rounded, base attenuate, margins obscurely undulate to crenate or distinctly obtusely dentate (3–7 teeth), translucent white or brown ciliate, both surfaces glabrous. Leaves of sterile branches, opposite, 4–8 pairs; petiole 4–15 mm long, entirely ciliate; blade to 1.5 × 1.5 cm, suborbicular or widely ovate to ovate, apex rounded, base cuneate to narrowly cuneate, margins crenate with 3–10 flat obscure teeth, translucent white or brown ciliate, upper surface sparsely pilose near the margin, green to pale green, lower surface sparsely pilose along the veins, greenish grey. Inflorescence 5- to 30-flowered cyme, surrounded by leaf-like bracts; pedicel 1–3 mm long, sparsely pilose. Bracteal leaves yellow during flowering, turning to greenish yellow after anthesis; petiole 1–3 mm long, entirely ciliate; blade 2–9 × 2–10 mm, flabellate, obdeltoid, spatulate, apex obtuse to subtruncate, base narrowly cuneate to cuneate, margins obscurely undulate to crenate or distinctly obtusely dentate, 2–7 teeth, sparsely translucent white or brown ciliate, both surfaces glabrous, greenish-grey. Flowers tetramerous, actinomorphic; sepals 4 (2 pairs), free, petaloid, 1 pair overlapping the other in bud, erect, yellow, 2–4 × 2–3 mm, widely obovate to widely subelliptic, glabrous, 3-veined, apex obtuse to truncate, slightly recurved to outside, persistent; petals absent; stamens 8, biseriate, ca. 2 mm long, shorter than sepal; filaments narrow conical, ca. 1.5 mm long; anthers yellow, 2-locular, ca. 0.5 mm long, longitudinally dehiscent; pistil 2-carpellate, semi-inferior, ovary 1-locular, ovules at 2 parietal placentae, styles 2, free, ca. 1 mm long, stigma round. Fruit a capsule, light green, glabrous, ca. 6 mm long, 2-lobed (horn shaped), lobes slightly unequal, dehiscent along the adaxial suture; seeds numerous, light brown, ellipsoid, with a raphe on one side, thick-walled, 935–1021 × 511–566 μm, seed surface covered with minute deciduous papillae, without tubercles.

#### Etymology.

The specific epithet of the new species refers to the distinctly larger size of the seeds compared with those of other members in the series *Pilosa*.

#### Vernacular name.

Cheon Ji Gwaeng I Nun (Korean pronunciation); 천지괭이눈 (Korean name), Tiān Chí Jīn Yāo (Chinese pronunciation); 天池金腰 (Chinese name)

#### Distribution.

*Chrysosplenium macrospermum* is only known from Changbaishan Mt. in Jilin Province of China, at an elevation of ca. 2,600 m. To date, only a few subpopulations with approximately 5,000 individuals have been discovered near Tianchi Crater Lake. In the absence of additional data, we presently score it as Data Deficient (DD) according to the IUCN Red List criteria (IUCN 2001).

#### Ecology.

*Chrysosplenium macrospermum* occurs in alpine tundra, where it grows in humid and semi-shaded areas near the Tianchi volcanic crater along with Papaver radicatum var. pseudoradicatum (Kitag.) Kitag., *Bistorta ochotensis* Kom., *Micranthes laciniata* (Nakai & Takeda) S. Akiyama & H. Ohba, *Sedum rosea* (L.) Scop., and *Pedicularis verticillata* L. The flowering period of this species is from late May to early July, and the fruiting period is from July to August.

#### Additional specimens examined (paratype).

China. Jilin: near Tianchi (Cheon-Ji in Korean) Crater Lake to Changbaishan Mt., Antu County, Changchun, 25 Apr. 2014, *D.K. Lee-2014001* (HHU), *D.K. Lee-2014002* (HHU), *D.K. Lee-2014003* (HHU), 42°01'44.80"N, 128°03'59.22"E, elev. 2610 m, 26 Jul. 2017, *KYI-2017002* (HHU), *KYI-2017003* (HHU), *KYI-2017004* (HHU), *KYI-2017005* (HHU), *KYI-2017006* (KB).

#### Notes.

The new taxon and *C. valdepilosum* exhibit a high degree of morphological similarity upon flowering (Fig. 3) but can be distinguished by several characters, including the size of the seed, the excrescence of the seeds, the developmental form of the sterile branch, and the hair type on the leaves of the sterile branch (Table 1). *Chrysosplenium macrospermum* occurs only in the vicinity of Tianchi Lake (elev. 2190 to 2610 m). It is the only species of the series Pilosa that grows in the vast Changbaishan Mt. region. The geographical distributions of other members of series Pilosa, including *C. valdepilosum* (endemic to Korea), do not overlap with that of *C. macrospermum*.

**Table 1.** Comparison of the key features of *Chrysosplenium macrospermum* and *C. valdepilosum*.

Character	*C. macrospermum*	*C. valdepilosum*
Root	thick, stout	filiform, rather soft
Sterile branch	arch-shaped	straight
upper surface of leaf	sparsely pilose near the margin	pilose
Flowering stem	1–3	1
hair type	sparsely pilose	pilose
color	Green	green and purple (lower part of stem)
branched	often branched	not branched
Seed		
size	length/width range 935–1021/511–566 μm	length/width range 578–758/409–589 μm
surface	smooth (without tubercles)	with tubercles

### Key to species of *Chrysosplenium* series *Pilosa* modified from Kim et al. (2018)

**Table:** 

1	Sepals white. Anthers dark red	**2**
–	Sepals yellow or greenish. Anthers yellow	**3**
2	Stamens longer than or equal to sepals. Ovary superior. Seeds with tubercles	***C. album***
–	Stamens shorter than sepals. Ovary subsuperior. Seeds smooth	***C. hebetatum***
3	Sterile branches often hypogeous, filiform, with bulbil at top	***C. maximowiczii***
–	Sterile branches epigeous without bulbil	**4**
4	Seeds without tubercles	**5**
–	Seeds with tubercles	**7**
5	Sterile branches arch-shaped. Flowering stem(s) 1–3, sometimes branched. Seeds 935–1021 × 511–566 μm	***C. macrospermum***
–	Sterile branches straight (not arch-shaped). Flowering stem 1, not branched. Seeds 528–785 × 369–704 μm	**6**
6	Leaves of sterile branches congested at distal end, with white variegated veins on upper surface	***C. flaviflorum***
–	Leaves of sterile branches distantly arranged, with silvery dotted upper surface	***C. epigealum***
7	Seed tubercles arranged on inconspicuous longitudinal ridges	**8**
–	Seed tubercles arranged on prominent longitudinal ridges	**10**
8	Leaves of sterile branches densely ciliate	***C. villosum***
–	Leaves of sterile branches rarely ciliate	**9**
9	Sterile branches branched (at least two times), ca. 30 cm long after fruiting. Leaves of sterile branches with silvery dots, upper surface glabrous. Bracteal leaves yellowish-green	***C. ramosissimum***
–	Sterile branches unbranched, less than 15 cm long after fruiting. Leaves of sterile branches without silvery dots, upper surface pilose. Bracteal leaves bright yellow	***C. valdepilosum***
10	Basal leaves persistent	**11**
–	Basal leaves withered before flowering	**13**
11	Sepals yellow. Stamens shorter than sepals	***C. sphaerospermum***
–	Sepals light green. Stamens equal to or longer than sepals	**12**
12	Stamens equal to or slightly longer than sepals. Ovary 1/2 or 1/3 inferior	***C. rhabdospermum***
–	Stamens longer than sepals. Ovary 1/4 inferior or nearly superior	***C. pseudopilosum***
13	Leaves of sterile branches distantly arranged after fruiting. Bracteal leaves golden yellow, yellowish-green or green at flowering	**14**
–	Leaves of sterile branches congested at distal end after fruiting. Bracteal leaves green	**15**
14	Leaves of sterile branches pilose. Bracteal leaves golden yellow at flowering	***C. aureobracteatum***
–	Leaves of sterile branches glabrous. Bracteal leaves yellowish-green to green at flowering	***C. pilosum***
15	Seeds ca. 720 × 640 μm, with ca. 18 ridges, densely papillate	***C. barbatum***
–	Seeds ca. 640 × 510 μm, with ca. 16 ridges, sparsely papillate	***C. fulvum***

## Supplementary Material

XML Treatment for Chrysosplenium macrospermum
